# Health behaviors among young adult cancer survivors and the importance of task self-efficacy

**DOI:** 10.1007/s11764-026-02017-1

**Published:** 2026-04-02

**Authors:** Brianna N. Leitzelar, Justin B. Moore, Greg Russell, Carmina G. Valle, David Victorson, Betina Yanez, Stacy D. Sanford, Mallory A. Snyder, Sofia F. Garcia, John M. Salsman

**Affiliations:** 1 School of Kinesiology, University of Minnesota-Twin Cities, 1900 University Ave SE, Minneapolis, MN 55455 USA; 2 Department of Implementation Science, Division of Public Health Sciences, Wake Forest University School of Medicine, 1 Medical Center Blvd., Winston-Salem, NC 27157 USA; 3 Department of Biostatistics and Data Science, Wake Forest University School of Medicine, 1 Medical Center Blvd., Winston-Salem, NC 27157 USA; 4 Department of Nutrition, UNC Gillings School of Global Public Health and School of Medicine, University of North Carolina at Chapel Hill, 2004, Michael Hooker Research Center , Chapel Hill, NC 27599 USA; 5 Department of Medical Social Sciences, Northwestern University Feinberg School of Medicine, 625 Michigan Ave., Chicago, IL 60611 USA; 6 The Robert H. Lurie Comprehensive Cancer Center of Northwestern University, 675 N. St. Clair, Chicago, IL 60611 USA; 7 Osher Center for Integrative Health at Northwestern University, Northwestern University Feinberg School of Medicine, 150 E. Huron St., Chicago, IL 60611 USA; 8 Department of Psychiatry and Behavioral Sciences, Northwestern University Feinberg School of Medicine, 676 North Saint Clair St., Chicago, IL 60611 USA; 9 Office of Research, The University of Chicago, 6054 S. Drexel Avenue, Chicago, IL USA; 10 Department of Social Sciences and Health Policy, Division of Public Health Sciences, Wake Forest University School of Medicine, 1 Medical Center Blvd., Winston-Salem, NC 27157 USA

**Keywords:** Health behavior, Survivorship, Self-efficacy, Young adults

## Abstract

**Purpose:**

To examine the prevalence and predictors of adhering to smoking, diet, exercise, and alcohol guidelines among young adult cancer survivors (cases) and matched controls without a history of cancer (controls) within 5 years post-treatment.

**Methods:**

Cases (*N* = 335, mean age = 31.8 years, women = 68%) and age-, education-, sex-, and partner-status matched controls (*N* = 335) from a national online research panel completed measures assessing demographic characteristics, health behaviors (smoking, diet, exercise, alcohol), and task self-efficacy. Conditional logistic regression estimated the difference in health behaviors and self-efficacy between cases and controls.

**Results:**

Rates of engagement in health behaviors were suboptimal across groups (Cases: 63% = abstaining from cigarettes or tobacco products daily, 29% =  ≥ 5 servings of fruits and vegetables/day (FV intake), 55% = moderate to vigorous exercise for > 30 min for ≥ 5 days/week, 70% = abstaining from alcohol; Controls: 70%, 20%, 39%, 70%, respectively). Compared to controls, cases were less likely to avoid smoking (OR = 0.70, *p* = .04), more likely to report meeting FV intake (OR = 1.63, *p* = .01) and exercise (OR = 1.85, *p* < .001) guidelines and engaged in similar rates of abstaining from alcohol (*p* = 0.48). Reporting high task self-efficacy was associated with 3.7 to 20.2 times higher odds for meeting recommendations for healthy lifestyle behaviors, regardless of cancer history.

**Conclusions:**

Reported engagement in health behaviors was low, particularly for diet and exercise, and young adult cancer survivors were more likely to report smoking compared to controls. Task self-efficacy was strongly associated with meeting recommendations.

**Implications for Cancer Survivors:**

Overall engagement in healthy behaviors was suboptimal and interventions that target task self-efficacy may support adherence to healthy lifestyle behaviors.

**Supplementary Information:**

The online version contains supplementary material available at https://doi.org/10.1007/s11764-026-02017-1.

## Introduction

There are more than 2 million cancer survivors alive in the U.S. who were diagnosed as adolescents or young adults (i.e., 15–39 years) [1, 2]. The first five years following cancer treatment is a critical time for cancer survivors as they transition from active treatment to survivorship. Not only does cancer diagnosis and treatment interrupt typical developmental milestones during young adulthood, (e.g., gaining independence, identity development, and career development [3]) young adult cancer survivors are at risk for significant post-treatment physical and mental health challenges [3–5]. Notably, young adult cancer survivors are at increased risk of cancer recurrence and second primary cancers within the first 5 years following treatment [6, 7] and are at higher risk for cardiovascular disease and other chronic conditions (e.g., endocrine diseases, musculoskeletal disorders) compared to their peers without a history of cancer [8, 9]. Engaging in healthy lifestyle behaviors, such as avoiding smoking, consuming a healthy diet, being physically active, and/or limiting alcohol use may mitigate the risk for adverse cancer [10, 11], morbidity [12], and mortality [13, 14] outcomes among post-treatment cancer survivors. Young adult cancer survivors may, therefore, be more motivated than their peers without a history of cancer to engage in healthy lifestyle behaviors [15, 16]. It may also be the case that engagement in unhealthy lifestyle behaviors, particularly smoking and alcohol use, may serve as an avoidant coping mechanism during survivorship [17, 18].

Supporting survivors in adopting, re-initiating, or maintaining healthy lifestyle behaviors is a core component of survivorship care planning [19–21]. Similar recommendations for healthy lifestyle behaviors are endorsed by organizations such as the American Cancer Society (ACS) [22, 23], the American Society of Clinical Oncology (ASCO) [24] and the World Cancer Research Fund/American Institute for Cancer Research (WCRF/AICR) [25]. Recommendations include avoiding tobacco products altogether, eating a healthy diet (e.g., 5–9 servings of fruits and vegetable, several servings of whole grains, avoiding processed foods and sugar-sweetened beverages, and avoiding red and processed meats daily), being physically active (e.g., 150–300 min of moderate-intensity aerobic physical activity per week), and avoiding alcohol. Prevalence rates for adhering to healthy lifestyle recommendations vary by behavior, with recent evidence demonstrating modest adherence to recommendations among cancer survivors between the ages of 18–44 years, including 17.3% for fruit and vegetable intake (FV intake), 50.7% for exercise, 69% for not smoking, and 76.9% for avoiding excessive alcohol intake [26]. Although this provides insight into current rates of adherence to health behaviors among primarily young adult cancer survivors, understanding how adherence compares to similarly-aged adults without a history of cancer would further our understanding of the unique context of cancer survivorship.

Controlled comparison studies investigating healthy lifestyle behaviors among young adult cancer survivors is somewhat limited. Previous studies demonstrated young adult cancer survivors engage in higher rates of smoking and similar rates of healthy diet (i.e., fruit and vegetable intake) and alcohol use (i.e., binge drinking) behaviors compared to their peers without a history of cancer [27–30]. Results for physical activity behavior are less consistent. Tai et al. (2012) found young adult cancer survivors had higher rates of physical inactivity compared to controls without a history of cancer [27], while three other studies found similar rates of meeting aerobic physical activity guidelines (e.g., 150 min of moderate-to-vigorous intensity aerobic physical activity per week [22]) between young adult cancer survivors and controls [29–31]. These studies also show factors such as social support, access to healthcare, and psychological well-being are associated with health behaviors among young adult cancer survivors. It is important to note that most participants in previous case–control studies were long-term survivors (10 years or more) [27–29, 31], thus the prevalence of engaging in healthy behaviors during the first five years post-treatment among young adult cancer survivors is not well-known.

There is also increasing interest in understanding behavioral mechanisms for engaging in health behaviors. Self-efficacy, or one’s belief in their ability to succeed in specific contexts [32, 33], is a behavioral construct that cuts across most behavioral theories. In the health behavior context, self-efficacy is often considered in general (e.g., one has control over their own health behaviors), in relation to specific tasks (e.g., belief in one’s ability to avoid smoking), or in relation to overcoming barriers to engaging in a behavior (e.g., belief in ability to overcome barriers to exercise). Self-efficacy (task and/or barrier) is associated with smoking cessation [34, 35], higher fruit and vegetable consumption [35], more physical activity [34–36], and lower alcohol use [35] among adult cancer survivors between six weeks and one year post-treatment. High self-efficacy is thought to lead to higher behavioral engagement because high self-efficacy leads individuals to exert more resources and effort towards their goals, stimulates a more positive mindset, and allows individuals to persist in the face of challenges [32, 33]. Given that self-efficacy has been shown to predict health behavior among adult cancer survivors [34–36], extending this work to young adult cancer survivors is a logical next step.

The goal of this analysis was to better understand the patterns and predictors of young adult cancer survivors’ engagement in behavioral risk factors for cancer. Accordingly, we had the following objectives: (1) determine the prevalence of meeting recommendations for healthy lifestyle behaviors among young adult cancer survivors compared to matched controls without a history of cancer and if the prevalence differed based on time since treatment for young adult cancer survivors; (2) determine sociodemographic predictors of meeting recommendations for healthy lifestyle behaviors among young adult cancer survivors compared to matched controls; and (3) to determine if task self-efficacy predicts behavior among young adult cancer survivors compared to matched controls. By leveraging a controlled comparison design, we were uniquely positioned to examine the differential engagement in healthy behaviors that is characteristic of the unique development phases of young adulthood compared to engagement in healthy behaviors that may be catalyzed by a cancer diagnosis as a young adult cancer survivor. Further, our inclusion of a matched group of controls without a history of cancer allows us to make more precise comparisons relative to previous studies [27, 29, 37, 38].

## Materials and methods

### Study design and study setting

This was a secondary analysis using data from a case–control study of young adult cancer survivors (cases) and matched controls [39]. Participants were recruited and consented through Toluna, an Internet survey company (https://tolunacorporate.com/our-solutions/brand-and-campaign/custom-research-inc-qual/) with a global reach. Toluna is a web-based platform designed to facilitate internet-based research, including recruitment, consenting, and compensation. Toluna maintains databases of individuals who opt-in to be contacted for internet-based research opportunities. Participants receive reward points for their participation, which can be redeemed for a variety of incentives. The present study received ethics approval by the Northwestern University Institutional Review Board and participants completed electronic informed consent prior to completing the survey.

Young adult cancer survivors have lower rates of participation in research compared to other age groups [40]. The use of an online research panel is aligned with previous work from two large-scale efforts funded by the National Institutes of Health [41, 42], demonstrating online research panels to be an efficient and valid means of data collection for clinical and non-clinical samples. Toluna facilitated our reach to a large U.S. sample of young adult cancer survivors and individuals matched on age, education, partner status, and sex without a history of cancer.

### Participant eligibility and recruitment

Toluna managed recruitment, screening, and enrollment for the present study. Toluna sent e-mail invitations to potential participants from their databases of US community-dwelling adults. All interested individuals were screened using an online self-report survey to assess eligibility prior to enrolling in the study. Eligibility across all participants included access to a secure internet connection and the ability to read and understand English. Inclusion criteria specific to young adult cancer survivors included any adult between 18 and 39 years, with a diagnosis of cancer (except for basal cell skin carcinoma) who were within 0 to 60 months of completing curative intent treatment for their cancer. Exclusion criteria for young adult cancer survivors included recurrent cancer diagnosis, history of multiple primary cancers, and receipt of palliative or hospice care. Recruitment occurred across 14 months from 2010–2012. This dataset remains relevant, given the rise in early onset cancers and the growing number of young adult cancer survivors (over 2 million), [1, 2], recommendations for health behavior engagement have remained similar since the collection of this data [22, 43], and examining factors related to health behavior engagement are meaningful for understanding potential pathways for future research.

A total of 30,520 young adult cancer survivors accessed the study surveys, and of those, 3,944 were eligible (12.9%). To capture variability in the early survivorship period [44], young adult cancer survivors were also stratified across three groups based on time since treatment (0–12, 13–24, and 25–60 months). Approximately equal numbers of participants within each time-since-treatment group were recruited in order to approximate potential changes over time. Controls were eligible if they did not have a cancer history and shared the same age, education, sex, and partner status of a young adult cancer survivor participant. Of the 16,333 controls who accessed the survey, 2,383 were eligible and matched with a young adult cancer survivor group participant (14.6%).

Toluna incorporates several in-field quality checks to reduce the potential for fraudulent data and enhance data integrity. The criteria for potentially fraudulent entries included more than 20 items skipped, short completion times (i.e., less than one third of median response time), and straight-line responding (i.e., selecting the same response for a single questionnaire) were removed from the data set. We also removed data from young adult cancer survivor respondents who did not report an identifiable diagnosis and data from controls who reported a history of cancer in their health history questionnaire. More details regarding data quality control and fraud prevention can be found at https://tolunacorporate.com/ts-quality-center/. Of the 6,327 that were eligible (*n* = 3,944 cases, n = 2,383 controls), we removed suspicious responses from 5,657 respondents (n = 3,609 cases, n = 2,048 controls) respondents. The total sample was 670 (10.60% of eligible respondents), or 335 per group. Participant demographics and a detailed breakdown of cancer characteristics for the final sample are provided in Tables 1 and 2.

**Table 1:** Baseline patient demographics and clinical characteristics

Characteristic ^a^	Overall	Cases	Controls	*p*-value
	(*n* = 670)	(*n* = 335)	(*n* = 335)	
Sex, female ^b^	458 (68.4)	229 (68.4)	229 (68.4)	NA
Age, y ^b^	31.8 (5.4)	31.8 (5.4)	31.8 (5.4)	NA
Ethnicity				0.029
Yes, Hispanic	51 (7.6)	18 (5.4)	33 (9.9)	
No, not Hispanic	613 (91.6)	315 (94.3)	298 (89.0)	
Decline to answer	5 (0.7)	1 (0.3)	4 (1.2)	
Race				0.15
White	538 (80.4)	281 (84.1)	257 (76.7)	
Black/African American	41 (6.1)	17 (5.1)	24 (7.2)	
Asian/Pacific Islander	46 (6.9)	20 (6.0)	26 (7.8)	
American Indian/Alaska Native	7 (1.0)	5 (1.5)	2 (0.6)	
Mixed racial background	24 (3.6)	8 (2.4)	16 (4.8)	
Other	7 (1.0)	2 (0.6)	5 (1.5)	
Decline to answer	6 (0.9)	1 (0.3)	5 (1.5)	
Education ^b^				NA
Some high school	10 (1.5)	5 (1.5)	5 (1.5)	
High school graduate	80 (12.0)	40 (12.0)	40 (11.9)	
Some college	172 (25.7)	86 (25.7)	86 (25.7)	
College graduate	270 (40.4)	135 (40.4)	135 (40.4)	
Some grad school	40 (6.0)	20 (6.0)	20 (6.0)	
Graduate school	97 (14.5)	48 (14.4)	49 (14.6)	
Employment status				0.23
Employed full-time	361 (53.9)	192 (57.3)	169 (50.4)	
Employed part-time	63 (9.4)	27 (8.1)	36 (10.7)	
Self-employed	34 (5.1)	15 (4.5)	19 (5.7)	
Looking for work	54 (8.1)	26 (7.8)	28 (8.4)	
No longer looking for work	17 (2.5)	9 (2.7)	8 (2.4)	
Retired	7 (1.0)	6 (1.8)	1 (0.3)	
Student	52 (7.8)	26 (7.8)	26 (7.8)	
Homemaker	82 (12.2)	34 (10.1)	48 (14.3)	
Partner Status ^b^				NA
Married	376 (56.1)	188 (56.1)	188 (56.1)	
Single, never married	190 (28.4)	95 (28.4)	95 (28.4)	
Divorced	38 (5.7)	19 (5.7)	19 (5.7)	
Living with partner	46 (6.9)	23 (6.9)	23 (6.9)	
Separated	20 (3.0)	10 (3.0)	10 (3.0)	
Income				< 0.001
Less than $15,000	54 (8.1)	23 (6.9)	31 (9.3)	
$15,000 to $24,999	50 (7.5)	22 (6.6)	28 (8.4)	
$25,000 to $34,999	64 (9.6)	28 (8.4)	36 (10.7)	
$35,000 to $49,999	103 (15.4)	43 (12.8)	60 (17.9)	
$50,000 to $74,999	132 (19.7)	55 (16.4)	77 (23.0)	
$75,000 to $99,999	88 (13.1)	41 (12.2)	47 (14.0)	
$100,000 to $124,999	64 (9.6)	43 (12.8)	21 (6.3)	
$125,000 to $149,999	36 (5.4)	27 (8.1)	9 (2.7)	
$150,000 to $199,999	24 (3.6)	14 (4.2)	10 (3.0)	
$200,000 to $249,999	23 (3.4)	21 (6.3)	2 (0.6)	
$250,000 or more	12 (1.8)	8 (2.4)	4 (1.2)	
Decline to answer	20 (3.0)	10 (3.0)	10 (3.0)	
Self-efficacy				
Smoking, % high	487 (72.9)	236 (70.9)	251 (74.9)	0.26
Diet, % high	277 (41.3)	145 (43.3)	132 (39.4)	0.35
Physical Activity, % high	298 (44.5)	158 (47.2)	140 (41.8)	0.19
Alcohol Consumption, % high	514 (76.8)	244 (73.1)	270 (80.6)	0.02

^a^ Continuous measures are presented as mean and standard deviation; categorical measures are presented as frequency and proportion. “Decline to answer” responses were not included in testing for group differences

FV Intake = Fruit and vegetable intake

^b^ Age, education, sex, and partner status were variables used to match cases and controls

**Table 2:** Clinical characteristics for young adult cancer survivors

	*N* = 335	
Characteristic	*N*	%
Cohort		
0–12 months posttreatment	120	35.8
13–24 months posttreatment	102	30.4
25–60 months posttreatment	113	33.7
ECOG Performance Status		
Normal activity, without symptoms	181	54
Some symptoms, not requiring bed rest	127	37.9
Require bed rest for < 50% of waking day	24	7.2
Require bed rest for > 50% of waking day	3	0.9
Cancer Type		
Breast	80	23.9
Gynecologic (cervical, uterine, ovarian)	54	16.1
Melanoma	37	11.0
Lung	23	6.9
Colorectal	21	6.3
Thyroid	21	6.3
Testicular	20	6.0
Hematologic (NHL, Hodgkin, leukemia)	17	5.1
Stomach	12	3.6
Hepatobiliary (liver, pancreas, bile duct)	11	3.3
Head and neck	10	3.0
Bone and sarcomas (soft tissue and bone)	10	3.0
Esophageal	7	2.1
Urinary bladder, kidney, renal pelvis	7	2.1
Brain and central nervous system	5	1.5
Stage		
Local	225	67.4
Regional	83	24.9
Distal	15	4.5
Unclear	11	3.3
Missing ^a^	1	
Treatment ^b^		
Surgery, % yes	299	89.3
Chemotherapy, % yes	221	66.0
Radiation, % yes	174	51.9

^a^ participants could report more than one treatment

^b^ not included in frequence calculation

### Procedures

Eligible individuals completed electronic consent on the Toluna platform prior to initiating the survey. Surveys assessed demographic, medical, health behavior, and psychosocial variables. Young adult cancer survivors also completed questions to assess their cancer history (e.g., diagnosis, treatment, stage). Participants who completed the survey were eligible for points to use towards rewards (e.g., gift cards, cash, donations) through the Toluna platform.

### Measures

The following were included in the present analysis:

- Demographic characteristics and medical history

Demographic characteristics included age in years (calculated from birth year), education level, employment status, ethnicity (of Hispanic origin), sex (female/male), marital status, income level, and race. Medical history (young adult cancer survivors only) included date of cancer diagnosis, primary site, date of last treatment(s) (surgery, radiation, chemotherapy), and if their cancer spread to lymph nodes or another part of the body. Time since treatment was calculated based on date of most recent treatment and date of survey completion and classified by time since treatment (0–12 months, 13–24 months, 25–60 months). Cancer stages included local, regional, distal, and unclear. Young adult cancer survivors also reported their ECOG performance status levels (normal activity without symptoms, some symptoms without need for bed rest, require bed rest for < 50% of day, require bed rest for > 50% of day, unable to get out of bed).

- Health Behaviors

Adherence to health behaviors was assessed using study-specific items adapted from ACS guidelines [22, 23, 43]. Participants reported their frequency (never, rarely, couple times a week, daily, more than once a day) of using cigarettes or tobacco products (cigarettes/tobacco use), intake of 5–9 servings of fruits and vegetables (FV intake), and alcohol use. Participants also reported the number of times per week they engaged in at least 30 min of moderate and strenuous exercise. Responses were used to classify participants as meeting or not meeting recommendations based on the ACS guidelines (Table S1, Online Resource 1). Meeting recommendations included never using cigarettes or tobacco products (cigarette/tobacco use), daily or more than daily consumption of 5–9 servings of fruits and vegetables (FV Intake), reporting 5 or more days per week of moderate or strenuous exercise (exercise), and never consuming alcoholic beverages (alcohol use).

- Self-efficacy

Task self-efficacy was measured using study-specific items developed to align with recommendations for health behaviors. Four questions assessed confidence in abstaining from cigarettes or tobacco products, maintaining a healthy diet, maintaining a physically active lifestyle, and drinking in moderation or abstaining from drinking altogether (Table S2, Online Resource 1) using a 5-point Likert scale (not at all, a little bit, somewhat, quite a bit, very much). Participants were classified as high (quite a bit/very much) or low (somewhat, a little bit, not at all) self-efficacy for each of the four behaviors.

### Data analysis

All data were summarized using descriptive statistics to characterize the matched sample. An a priori power calculation revealed a sample of 670 (N = 335 per group) was needed to adequately detect small effect sizes (Cohen’s d = 0.2 to 0.3), considering an alpha level of 0.05 and 80% statistical power for the original study. Although no additional power calculation was conducted due to the secondary nature of the present analysis, the final sample size (two groups of N = 335) is comparable to or larger than prior case–control studies examining similar outcomes. Baseline categorical characteristics were compared between cases and controls using Fisher’s exact test to describe the distribution of participant characteristics and demonstrate the effectiveness of matching. These comparisons were intended for descriptive purposes only and were not used to support inferential conclusions.

All primary analyses evaluating differences in health behaviors between young adult cancer survivors and matched controls accounted for the 1:1 matched case–control design using conditional logistic regression, with matching on age, education, sex, and partner status incorporated by design. To determine prevalence of meeting healthy lifestyle behaviors between cases and controls (objective 1), odds ratios and 95% confidence intervals were estimated for meeting recommendations for cigarette/tobacco abstinence, FV intake, exercise, and alcohol use. Similar models investigated the variation in prevalence rates by time since diagnosis group, using case/control status and time-since-diagnosis group as predictors.

Additional regression models were used to evaluate the association between sociodemographic factors (age per 5 years, education, marital status, and employment status) and each health behavior outcome (objective 2) and, separately, behavior specific task self-efficacy and each health behavior outcome (objective 3). These models included case/control status and task self-efficacy as predictors. Interaction terms between case/control status and self-efficacy were tested and removed from final models when not statistically significant. Model assumptions were assessed through examination of model convergence and stability; given the limited number of predictors and absence of highly correlated covariates, no evidence of multicollinearity was observed. All analyses were conducted using SAS version 9.4 (Cary, NC, USA). Statistical significance was defined as *p* < 0.05.

## Results

### Participants

Included participants were mainly female (68.4%), non-Hispanic (91.6%), White (80.4%), well-educated (60.9% college graduates or higher), and married (56.1%) (Table 1). There were no significant differences in demographic characteristics with the exception of ethnicity and income level (*p* < 0.05). A smaller proportion of cases reported Hispanic ethnicity and high incomes compared to controls. Young adult cancer survivors were primarily diagnosed with breast (23.9%) or gynecologic (16.1%) cancers or melanoma (11%). Table 2 reports the full cancer-related characteristics for cases.

### Prevalence of engaging in healthy lifestyle behaviors

Prevalence rates and odds ratios (OR) by case/control status overall and by time since treatment group are presented in Table 3. Young adult cancer survivors were 30% less likely to abstain from cigarettes or tobacco use (OR: 0.70, 95% CI = 0.49, 0.99; *p* = 0.044), 63% more likely to report meeting FV intake (OR: 1.63, 1.13, 2.33; *p* = 0.0081), and 85% more likely to report meeting exercise (OR: 1.85, 95% CI = 1.36, 2.53) recommendations compared to controls. Prevalence rates for alcohol use were similar between young adult cancer survivor and controls overall (OR: 0.80, 95% CI: 0.54, 1.19, *p* = 0.27).

**Table 3:** Prevalence rates and odds ratios for adhering to healthy lifestyle behavior among young adult cancer survivors and matched controls without a history of cancer

		Cigarette/Tobacco Use	FV Intake	Exercise	Alcohol Use
Overall (*n* = 670)	Cases	62.7%	28.7%	54.3%	70.7%
	Controls	69.6%	19.7%	38.8%	70.1%
	OR (95% CI)	0.70 (0.49, 0.99)	1.63 (1.13, 2.33)	1.85 (1.36, 2.53)	0.80 (0.54, 1.19)
	*p*	0.044	0.0081	< 0.001	0.27
0–12 months (*n* = 120)	Cases	65.0%	29.2%	48.3%	75.8%
	Controls	74.2%	20.8%	35.8%	74.2%
	OR (95% CI)	0.67 (0.35, 1.25)	1.44 (0.79, 2.63)	1.50 (0.90, 2.51)	1.21 (0.66, 2.22)
	*p*	0.21	0.23	0.12	0.54
13–24 months (*n* = 102)	Cases	58.8%	32.4%	62.7%	61.8%
	Controls	64.7%	15.7%	40.2%	68.6%
	OR (95% CI)	0.74 (0.39, 1.38)	2.36 (1.17, 4.78)	2.71 (1.47, 5.01)	0.60 (0.32, 1.14)
	*p*	0.35	0.017	0.0014	0.12
25–60 months (*n* = 113)	Cases	63.7%	25.0%	53.1%	73.5%
	Controls	69.0%	22.1%	40.7%	67.3%
	OR (95% CI)	0.76 (0.42, 1.38)	1.06 (0.55, 2.01)	1.71 (1.001, 2.94)	1.22 (0.70, 2.11)
	*p*	0.37	0.86	0.050	0.48

FV Intake = Fruit and vegetable intake

Results were relatively consistent when stratified by time since treatment group. Observed differences between young adult cancer survivors and controls for smoking rates were consistent regardless of time-since-treatment group (*p* > 0.05). Young adult cancer survivors were more likely to meet FV intake and exercise recommendations across time since treatment groups, though this was most pronounced in the 13–24 month group. In fact, case/control comparisons were only significant in the 13–24 month group, with results demonstrating that young adult cancer survivors were 2.3 and 2.7 times more likely to meet diet and physical activity recommendations, respectively, compared to controls. Similar to the overall findings, differences in alcohol use between cases and controls were not present when stratified by time since treatment group (*p* > 0.05).

### Sociodemographic predictors

There were no significant associations between any of these variables and any behavioral outcome. Results from the analyses can be found in Online Resource 1 (Table S3).

### Self-efficacy as a predictor

Task self-efficacy was significantly associated with adherence to all behaviors measured (i.e., tobacco/cigarette use, FV intake, exercise, and alcohol use) (Table 4). There was no significant interaction between case/control status and self-efficacy for any behavior (Table S4, Online Resource 1). Participants with high self-efficacy for abstaining from cigarette or tobacco use were 20.20 times more likely to report never using cigarettes or tobacco compared to those with low self-efficacy (95% CI = 7.30, 55.60, *p* < 0.001). High self-efficacy for maintaining a healthy diet was associated with a 6.78-fold increase in consuming 5 to 9 servings of fruits and vegetables at least daily (95% CI = 3.39, 13.50, *p* < 0.001) and high self-efficacy for maintaining a physically active lifestyle was associated with a 6.19-fold increase in meeting physical activity guidelines (95% CI = 3.54, 10.80, *p* < 0.001). Participants reporting high self-efficacy for drinking in moderation or abstaining from alcohol were 3.72 times more likely to report never or rarely using alcohol (95% CI = 2.00, 6.92, *p* < 0.001).

**Table 4:** Odds ratios for adhering to healthy lifestyle behavior recommendations by case/control status and level of behavior-specific self-efficacy

Outcome	Covariate	Odds Ratio	95% Confidence Intervals		*p*-value
Cigarette/Tobacco Use	Case/Control^a^	0.67	0.40	1.12	0.12
	Self-Efficacy^b^	20.20	7.30	55.60	< 0.001
Diet	Case/Control^a^	1.78	1.14	2.77	0.011
	Self-Efficacy^b^	6.78	3.39	13.50	< 0.001
Exercise	Case/Control^a^	1.99	1.36	2.93	< 0.001
	Self-Efficacy^b^	6.19	3.54	10.80	< 0.001
Alcohol Use	Case/Control^a^	1.14	0.79	1.65	0.48
	Self-Efficacy^b^	3.72	2.00	6.92	< 0.001

FV Intake = Fruit and vegetable intake

^a^ reference: young adult cancer survivors vs. healthy controls

^b^ reference: high vs. low

## Discussion

This is the first study of early post-treatment young adult cancer survivor survivors (0–5 years) to examine reports of, and confidence in, adhering to healthy lifestyle behaviors compared to matched peers without a history of cancer. The ACS regularly publishes recommended guidelines for health behaviors to facilitate recovery from cancer, reduce the risk of recurrence, and promote quality of life [22, 23, 45]. Our results demonstrate that young adult cancer survivors within five years of treatment were less likely to avoid smoking, were more likely to consume 5–9 servings of fruits and vegetables and exercise, and engaged in similar levels of alcohol use compared to their peers without a history of cancer, though rates overall were suboptimal across our sample. Sociodemographic variables did not predict self-reports of health behaviors, but we found that task self-efficacy was strongly associated with self-reported health behavior regardless of one’s cancer history.

Our findings support and extend previous findings regarding smoking and alcohol behaviors among young adult cancer survivors [27, 29]. Previous studies focused on health risk behaviors and classified participants based on their rates of current smoking and binge drinking behaviors [27, 29]. Rates of binge drinking were not significantly different and ranged from 7.7–20.3% depending on cancer history [27–29] and sex [29]. Smoking rates were significantly higher among young adult cancer survivors (27–37%) than controls (13–26%) [27–30], though Warner and colleagues (2016) showed differences were only present among female young adult cancer survivors [29] and Choi and associates (2023) showed differences among only male young adult cancer survivors [30]. Unique to our study is a focus on adherence to health-promoting behaviors, such as abstaining from alcohol and tobacco, rather than the focus on health-risk behaviors seen in previous studies. Our results demonstrate that 63% of young adult cancer survivors and 70% of matched controls reported avoiding cigarette and tobacco products and approximately 70% of our sample refrained from alcohol use. Although it seems most young adults regardless of cancer history do not engage in alcohol or smoking behaviors, intervening to address and reduce alcohol use and smoking among those who do remains important for optimal health [25, 46].

Although young adult cancer survivors were more likely than their matched peers without a history of cancer to report meeting FV intake and exercise guidelines in our sample, the prevalence of meeting recommendations was low overall (20–54%). Our findings do not align with most previous research, which found young adult cancer survivors and controls had similar physical activity and FV intake behaviors [27–31] or that young adult cancer survivors were less active than controls [27]. The present sample were restricted to five years post-diagnosis, whereas young adult cancer survivors in previous research were 10 years or more post-diagnosis. One possible explanation for these differences is the ‘teachable moment [15, 16], or a period of time following a specific event that may motivate one to adopt healthy behaviors. It could be that young adult cancer survivors more proximal to their treatment are more motivated to *initiate* healthy eating patterns, such as consuming 5–9 fruits and vegetables per day, and *maintaining* healthy behaviors may be challenging when they are more distal from their diagnosis. However, we should note that our data may not support this hypothesis for smoking and alcohol behaviors, which could be because some survivors engage in unhealthy coping using tobacco and/or alcohol [17, 18]. Reaching the 5-year mark post-treatment is a significant event in cancer survivorship [19], whereby the risk for cancer recurrence significantly declines [6, 7]. Understanding the long-term patterns of motivation following diagnosis and treatment among young adult cancer survivors is an important area of future study.

The lack of association between sociodemographic factors and health behaviors in our sample was surprising, although the literature is somewhat limited in this area. Previous studies demonstrated low socioeconomic status (income, education, financial difficulties) was associated with low physical activity levels and less hard alcohol use [47] among young adult cancer survivors. Sex has been associated with adhering to FV intake and alcohol use guidelines, but not physical activity guidelines [47]. Werner and associates (2016) stratified their analyses by sex and found that younger age was associated with higher rates of smoking and binge drinking in females; whereas younger age was associated only with binge drinking in males. For males only, being uninsured was associated with a higher risk for current smoking. The lack of association in the current sample may reflect the fact that young adult cancer survivors and controls were matched on many of these factors (i.e., age, sex, education, marital status), or the homogeneity of the sample (i.e., high education and income levels, predominantly White), thus removing some variability for analysis. It could also be that cancer history is an important consideration when determining one’s likelihood of engaging in health behaviors regardless of sociodemographic factors.

This analysis is the first to confirm task self-efficacy is a powerful factor associated with adherence to health-promoting behaviors among young adult cancer survivors, although directionality cannot be determined by the present analysis. In one study of childhood and young adult cancer survivor survivors, general self-efficacy for engaging in healthy lifestyles in general was not shown to be associated with tobacco use, but was associated with lower engagement in unhealthy lifestyle behavior [48]. In contrast, we found that having high task self-efficacy for engaging in health-promoting behaviors was associated with a 3 to 20-fold increase in the likelihood of engaging in that specific behavior, including cigarette/tobacco use, FV intake, exercise, and alcohol use behaviors. Our findings align with results from studies of adult cancer survivors which also found moderate to strong associations between higher task self-efficacy and engaging in healthy behaviors [35, 36], supporting the idea that task-specific self-efficacy is important rather than general self-efficacy. However, most research is cross-sectional and it is possible that high rates of engaging in a behavior lead to high rates of confidence in performing the behavior. One longitudinal study in this area demonstrated that task-self efficacy predicted physical activity behavior 2 months later, supporting the theoretical temporal nature of these relationships.

Promoting adherence to healthy lifestyle behaviors aids in recovery from cancer treatment and supports longevity and quality of life among post-treatment cancer survivors [10–14]. The overall low prevalence of meeting healthy lifestyle recommendations, particularly FV intake and exercise, highlights the continued need for future efforts to understand effective strategies to promote health behavior engagement. It is important to acknowledge that there has been increasing attention on promoting healthy lifestyles following cancer treatment since the data for the present study were collected. Many NCI Comprehensive Cancer Centers have initiated cancer survivorship programs aiming to increase access to supportive programming throughout survivorship; and significant challenges still exist in this area, such as availability of healthcare providers with training and expertise in behavioral health and lack of insurance reimbursement for supportive services, and referral pathways to improve access to onco-primary care [49].

Healthcare providers have a unique opportunity to discuss engagement in healthy lifestyle behaviors, prompt behavioral changes, and facilitate access to resources. The findings from the present study may be used to begin important clinical conversations about current health behaviors and initiating behavioral changes. Our findings suggest that supporting task self-efficacy through social-cognitive strategies (e.g., setting and achieving small incremental goals, observing others’ success, receiving encouragement from social sphere, and managing emotions and building resilience) [32], may be a key strategy to improve adherence to healthy behaviors. This may be particularly salient for smoking and alcohol use behaviors, as our data showed that including self-efficacy in the models eliminated the differences we saw between young adult cancer survivors and controls. This suggests that one’s *confidence* in their ability to abstain from smoking or alcohol use may be more important for predicting smoking behavior than one’s history with cancer.

There are several strengths and limitations to this analysis. Leveraging the Toluna platform allowed us to reach a large, national sample of young adult cancer survivor survivors who are understudied in cancer research [50, 51]. We were also able to match young adult cancer survivors and controls on age, education, sex, and partner status, which allowed us to make more precise case–control comparisons. We can be more confident that the differences noted between cases and controls are due to their cancer history rather than sociodemographic factors. However, it is important to note that we were not able to include all sociodemographic factors such as race, ethnicity, or geographic location, which have been related to engagement in health-promoting behaviors in previous studies [29].

Although the use of an online panel allowed us to reach an understudied population, it also resulted in a study sample that was relatively affluent and limited in racial and ethnic diversity, which may overestimate the rate of health behavior engagement among young adult cancer survivors. The present data were collected several years ago, which may limit the generalizability to the current landscape. Although recommendations for healthy lifestyle behaviors and challenges to adhering to those recommendations are comparable, it remains possible that access to survivorship care, health and fitness apps, wearables, and/or associated behavioral interventions since these data were collected may inform interpretations and implications of our analysis in ways not fully captured here. We also note that, although direct comparisons are not possible due to methodological differences between studies, our prevalence rates are comparable to recently reported rates of health behavior engagement among adults aged 18–44 years [26]. Supporting sustained behavior change and disseminating effective interventions remain significant gaps for young adult cancer survivors [52]. Matching cases and controls on age, education, sex, and partner status reduced potential confounding of sociodemographic variables, but we did not measure other health conditions which may influence rates of behavioral engagement.

The removal of approximately 90% of survey responses with “suspicious” response patterns deserves further discussion. Rising challenges in digital health research, including fraudulent participants, or those who attempt to lie or hide their true identity in order to receive the study incentives, require rigorous criteria to detect fraudulent data to maintain data integrity [53, 54]. While necessary to ensure the integrity of the data, removing data may have introduced some selection bias. Suggestions for enhancing transparency in future publications include explicitly reporting criteria for detecting fraudulent responses and including rates of suspected fraudulent data.

Measurement bias may also be present in our results, given that health behaviors were assessed using self-report items focused on a single behavior within each domain (e.g., FV Intake for diet and exercise for physical activity) and the items used to measure self-efficacy were single-item study derived measures. It is also possible that dichotomizing into groups may overestimate the associations between case–control status or self-efficacy and adherence to health behaviors. Using single-item measures to assess behavior and self-efficacy in this study supported a pragmatic approach to reduce participant burden. Utilizing multi-item self-report measures or fitness trackers, such as the Food Frequency Questionnaire [55] (FFQ) for diet or accelerometry for physical activity [56], would allow the use of continuous data, thereby enhancing the precision of effect estimates in future studies. The cross-sectional analysis and lack of pre-morbid reports of health behavior engagement limits our ability to make causal statements, and longitudinal studies would provide further insight into engagement in lifestyle behaviors during the transition from active treatment to long-term cancer survivorship.

## Conclusion

Adherence to healthy lifestyle behaviors among adults aged 18–39 years, regardless of cancer history, was suboptimal, particularly for diet and physical activity behaviors. Sociodemographic factors did not appear to affect the association between case–control status and adherence to health-promoting behaviors, suggesting that history of cancer may be associated with better diet and physical activity and worse smoking behaviors among young adult cancer survivors compared to peers without a history of cancer, regardless of age, sex, marital status, or employment status. The finding that adherence rates were low in the combined sample highlights the need for interventions designed to increase adherence to health-promoting behaviors. Self-efficacy, a key behavioral factor, was strongly associated with meeting guidelines for healthy behaviors in our sample. This suggests that targeting task self-efficacy is likely to be a powerful intervention strategy for increasing adherence to healthy behaviors among young adults.

## Supplementary Information

Below is the link to the electronic supplementary material.

ESM 1 (DOCX 129 KB)

## Data Availability

De-identified data from this study may be available on reasonable request and with appropriate ethical approvals.
