# Supplementary material for: Health behaviors among young adult cancer survivors and the importance of task self-efficacy
Source: J Cancer Surviv. 2026 Apr 2;20(Suppl 2):1789–801. doi: 10.1007/s11764-026-02017-1 (PMC13744496; doi:10.1007/s11764-026-02017-1)
Supplement: Supplementary file 1 [file 11764_2026_2017_MOESM1_ESM.docx]

**Article Title:**	Health behaviors among young adult cancer survivors and the importance of behavior-specific self-efficacy

**Journal name:**	Journal of Cancer Survivorship

**Authors:**	Brianna N. Leitzelar^a^, Justin B. Moore^b^, Greg Russell^c^, Carmina G. Valle^d^, David Victorson^e, f, g^, Betina Yanez^e, f^, Stacy D. Sanford^e, f, h^, Mallory A. Snyder^i^, Sofia F. Garcia^e, f, h^, John M. Salsman^j^,

^a^ School of Kinesiology, University of Minnesota-Twin Cities, Minneapolis, MN, USA

^b^ Department of Implementation Science, Division of Public Health Sciences, Wake Forest University School of Medicine, Winston-Salem, USA;

^c^ Department of Biostatistics and Data Science, Wake Forest University School of Medicine, Winston-Salem, NC, USA

^d^ Department of Nutrition, UNC Gillings School of Global Public Health and School of Medicine, University of North Carolina at Chapel Hill, Chapel Hill, NC, USA

^e^ Department of Medical Social Sciences, Northwestern University Feinberg School of Medicine, Chicago, IL, USA

^f^ The Robert H. Lurie Comprehensive Cancer Center of Northwestern University, Chicago, IL, USA

^g^ Osher Center for Integrative Health at Northwestern University, Northwestern University Feinberg School of Medicine, Chicago, IL, USA

^h^ Department of Psychiatry and Behavioral Sciences, Northwestern University Feinberg School of Medicine, Chicago, IL, USA

^i^ Office of Research, The University of Chicago, Chicago, IL, USA

^j^ Department of Social Sciences and Health Policy, Division of Public Health Sciences, Wake Forest University School of Medicine, Winston-Salem, NC, USA

Corresponding author:

Brianna N. Leitzelar

email: leitz025@umn.edu

phone: (612) 624-8549

Online Resource Captions Online Resource 1. Supplementary tables summarizing the classifications for healthy lifestyle behaviors (Table S1), self-efficacy items (Table S2), odds ratios for adhering to healthy lifestyle behavior recommendations by case/control status and sociodemographic factor (Table S3), and interaction effects of case/control status by level of self-efficacy on adherence to health lifestyle behavior recommendations (Table S4).

**Table S1**. Classifications for healthy lifestyle behaviors.

| Behavior | Question | Definition |
| --- | --- | --- |
| Cigarette or Tobacco Use | “use of cigarettes or tobacco products” ^b^ | Meets recommendations:  abstaining from cigarettes or tobacco products |
|  |  | Does not meet recommendations:  any smoking |
| Fruit and Vegetable Intake | “I eat five to nine servings of fruits and vegetables” ^b^ | Meets recommendations:  5-9 servings of fruits and vegetables per day daily or more than daily |
|  |  | Does not meet recommendations:  5-9 servings of fruits and vegetables per day never, rarely, couple times a week |
| Exercise^a^ | “I engage in strenuous exercise for more than 30 minutes during my free time” and “I engage in moderate exercise for more than 30 minutes during my free time”^c^ | Meets recommendations:  moderate or vigorous exercise for 30 minutes for ≥5 days/week |
|  |  | Does not meet recommendations:  moderate or vigorous exercise for 30 minutes for <5 days/week |
| Alcohol Use | “I drink alcoholic beverages” ^b^ | Meets recommendations:  abstains from alcohol |
|  |  | Does not meet recommendations:  any consumption |
| ^a^ Although the American Cancer Society guidelines focus on physical activity recommendations (i.e., a minimum of 150 minutes of moderate-to-vigorous intensity physical activity per week),^21^ our items were specific to exercise. Exercise is a subset of physical activity behavior which focuses on choice-based physical activity during leisure-time, which does not capture other physical activity behaviors that contribute to achieving the guidelines such as occupational, transportation, and household/caregiving-related physical activities. | | |
| ^b^ participants reported frequency using the following response options: never, rarely, couple times a week, daily, and more than once a day | | |
| ^c^ participants reported the number of times per week | | |

| **Table S2**. Self-efficacy items. | |
| --- | --- |
| Behavior | Item |
| Cigarette or Tobacco Use | “I am confident in my ability to abstain from cigarette or use of tobacco products” |
|  |  |
| Fruit and Vegetable Intake | “I am confident in my ability to maintain a healthy diet” |
|  |  |
| Exercise | “I am confident in my ability to maintain a physically active lifestyle” |
|  |  |
| Alcohol Use | “I am confident in my ability to drink in moderation or abstain from drinking altogether” |
| Note. Items were rated on a 5-point Likert scale (not at all, a little bit, somewhat, quite a bit, very much). | |

| **Table S3.** Odds ratios for adhering to healthy lifestyle behavior recommendations by case/control status and sociodemographic predictor. | | | | |
| --- | --- | --- | --- | --- |
| **Outcome** | **Covariate** | ***p*-value** | **OR (95% CI)** |  |
| Cigarette/Tobacco Use | Case/Control | 0.048 | 0.70 (0.49, 0.997) |  |
|  | Age (per 5 years) | 0.59 |  |  |
|  | Case/Control | 0.051 | 0.70 (0.49, 1.002) |  |
|  | Sex | 0.78 |  |  |
|  | Case/Control | 0.045 | 0.70 (0.49, 0.99) |  |
|  | Education | 0.49 |  |  |
|  | Case/Control | 0.044 | 0.70 (0.49, 0.99) |  |
|  | Marital status | 0.93 |  |  |
|  | Case/Control | 0.064 | 0.72 (0.50, 1.02) |  |
|  | Employment Status | 0.55 |  |  |
| FV Intake | Case/Control | 0.011 | 1.60 (1.12, 2.31) |  |
|  | Age (per 5 years) | 0.21 |  |  |
|  | Case/Control | 0.010 | 1.61 (1.12, 2.31) |  |
|  | Sex | 0.17 |  |  |
|  | Case/Control | 0.012 | 1.60 (1.11, 2.30) |  |
|  | Education | 0.99 |  |  |
|  | Case/Control | 0.0077 | 1.64 (1.14, 2.36) |  |
|  | Marital status | 0.99 |  |  |
|  | Case/Control | 0.012 | 1.61 (1.11, 2.33) |  |
|  | Employment Status | 0.69 |  |  |
| Exercise | Case/Control | <0.001 | 1.87 (1.37, 2.56) |  |
|  | Age (per 5 years) | 0.32 |  |  |
|  | Case/Control | <0.0001 | 1.85 (1.35, 2.53) |  |
|  | Sex | 0.23 |  |  |
|  | Case/Control | <0.001 | 1.92 (1.40, 2.63) |  |
|  | Education | 0.15 |  |  |
|  | Case/Control | <0.001 | 1.87 (1.37, 2.56) |  |
|  | Marital status | 0.70 |  |  |
|  | Case/Control | <0.001 | 1.88 (1.37, 2.57) |  |
|  | Employment Status | 0.21 |  |  |
| Alcohol Use | Case/Control | 0.97 | 1.01 (0.72, 1.41) |  |
|  | Age (per 5 years) | 0.25 |  |  |
|  | Case/Control | 0.89 | 1.03 (0.73, 1.44) |  |
|  | Sex | 0.58 |  |  |
|  | Case/Control | 0.87 | 1.03 (0.74, 1.44) |  |
|  | Education | >0.99 |  |  |
|  | Case/Control | 0.93 | 1.02 (0.72, 1.43) |  |
|  | Marital status | 0.99 |  |  |
|  | Case/Control | 0.79 | 1.03 (0.73, 1.45) |  |
|  | Employment Status | 0.34 |  |  |
| FV Intake = Fruit and vegetable intake | | | | |

**Figure S1.** Odds ratios (OR) overall and by time since treatment group for (a) Cigarette/Tobacco Use, (b) FV Intake, (c) Exercise, and (d) Alcohol Use between young adult cancer survivors (YA) and healthy controls (HC). OR greater than 1 indicates YAs are more likely to adhere and OR less than 1 indicates YAs are less likely to adhere.

FV Intake = Fruit and vegetable intake

| **Table S4.** Interaction effects of case/control status by level of self-efficacy on adherence to healthy lifestyle behavior recommendations. | | | |
| --- | --- | --- | --- |
| **Outcome** | **Comparison** | **OR (95% CI)** | ***p*-value** |
| Cigarette/Tobacco Use | Cases: High vs. Low | 32.1 (7.72, 133.2) | 0.33 |
|  | Controls: High vs. Low | 14.5 (4.38, 48.3) |  |
|  | High SE: Cases vs. Controls | 0.80 (0.43, 1.49) |  |
|  | Low SE: Cases vs. Controls | 0.36 (0.09, 1.40) |  |
| FV Intake | Cases: High vs. Low | 6.24 (2.55, 15.2) | 0.77 |
|  | Controls: High vs. Low | 7.37 (3.01, 18.1) |  |
|  | High SE: Cases vs. Controls | 1.64 (0.80, 3.34) |  |
|  | Low SE: Cases vs. Controls | 1.93 (0.94, 4.00) |  |
| Exercise | Cases: High vs. Low | 5.46 (2.51,11.9) | 0.65 |
|  | Controls: High vs. Low | 6.96 (3.27,14.8) |  |
|  | High SE: Cases vs. Controls | 1.74 (0.87, 3.49) |  |
|  | Low SE: Cases vs. Controls | 2.22 (1.20, 4.08) |  |
| Alcohol Use | Cases: High vs. Low | 3.02 (1.37, 6.70) | 0.41 |
|  | Controls: High vs. Low | 4.88 (1.96, 12.1) |  |
|  | High SE: Cases vs. Controls | 1.01 (0.64, 1.61) |  |
|  | Low SE: Cases vs. Controls | 1.64 (0.64, 4.18) |  |
| FV Intake = Fruit and vegetable intake | | | |
